# Diverse hydrocarbon biosynthetic enzymes can substitute for olefin synthase in the cyanobacterium *Synechococcus* sp. PCC 7002

**DOI:** 10.1038/s41598-018-38124-y

**Published:** 2019-02-04

**Authors:** Cory J. Knoot, Himadri B. Pakrasi

**Affiliations:** Department of Biology, Washington University, St. Louis, Missouri 63130 USA

## Abstract

Cyanobacteria are among only a few organisms that naturally synthesize long-chain alkane and alkene hydrocarbons. Cyanobacteria use one of two pathways to synthesize alka/enes, either acyl-ACP reductase (Aar) and aldehyde deformylating oxygenase (Ado) or olefin synthase (Ols). The genomes of cyanobacteria encode one of these pathways but never both, suggesting a mutual exclusivity. We studied hydrocarbon pathway compatibility using the model cyanobacterium *Synechococcus* sp. PCC 7002 (S7002) by co-expressing Ado/Aar and Ols and by entirely replacing Ols with three other types of hydrocarbon biosynthetic pathways. We find that Ado/Aar and Ols can co-exist and that slower growth occurs only when Ado/Aar are overexpressed at 38 °C. Furthermore, Ado/Aar and the non-cyanobacterial enzymes UndA and fatty acid photodecarboxylase are able to substitute for Ols in a knockout strain and conditionally rescue slow growth. Production of hydrocarbons by UndA in S7002 required a rational mutation to increase substrate range. Expression of the non-native enzymes in S7002 afforded unique hydrocarbon profiles and alka/enes not naturally produced by cyanobacteria. This suggests that the biosynthetic enzyme and the resulting types of hydrocarbons are not critical to supporting growth. Exchanging or mixing hydrocarbon pathways could enable production of novel types of CO_2_-derived hydrocarbons in cyanobacteria.

## Introduction

Cyanobacteria are an ancient and highly diverse phylum of photosynthetic prokaryotes with the capability to reproduce using (sun)light and carbon dioxide (CO_2_) as their primary energy and carbon sources, respectively. Extant cyanobacteria are key players in the global carbon and nitrogen cycles and as primary producers in the open ocean^1,2^. It has been known since the late 1960s that cyanobacteria naturally synthesize branched and linear C_15_ to C_19_ alkanes and alkenes^3,4^. The total amount of hydrocarbon produced per cell is around 0.02–0.35 percent of dry cell weight (% DCW)^5,6^. Importantly, the carbon chain lengths of most cyanobacterial hydrocarbons are within the range of fuels used currently in jet (C_8_–C_16_) and diesel engines (C_9_–C_36_)^7,8^, and there is, accordingly, interest in developing these organisms as bio-production platforms for CO_2_-derived biofuels^9–13^. In cyanobacteria, alka/ene hydrocarbons most likely localize to the thylakoid and plasma membranes^14^ where they serve numerous important roles. Knock-out strains that are unable to produce alka/enes exhibit a variety of phenotypes including greatly decreased growth rates, increased cyclic electron flow in the photosystems, changes in cell morphology, and salt sensitivity^14–16^. Typically, the slower growth of the knockout strains is more pronounced at lower growth temperatures^15,17^. Although many of the impacts of hydrocarbons on cells are known, the exact mechanism by which they produce these effects *in vivo* remains enigmatic.

**Figure 1:**
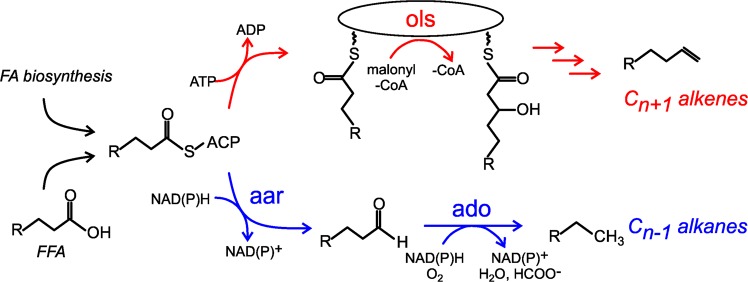
The two major alka/ene hydrocarbon biosynthetic pathways in cyanobacteria are the Ols (red) and Ado/Aar (blue) pathways. Fatty acid (FA) biosynthesis or activation from free fatty acids (FFA) provides acyl-ACP substrates for both pathways.

Two distinct biosynthetic pathways for alka/enes have been characterized in cyanobacteria^18,19^ (Fig. 1). The substrates for these native pathways are fatty acids (FAs) covalently linked to acyl carrier protein (fatty acyl-ACPs) produced by the FA synthase pathway^20^. One type of hydrocarbon biosynthesis pathway relies on two enzymes to convert acyl-ACP to alkane (Fig. 1, blue path). The first enzyme, acyl-ACP reductase (Aar), reduces substrate to the corresponding free fatty aldehyde using NAD(P)H as a cofactor^18,21^. The second enzyme, aldehyde deformylating oxygenase (Ado), uses molecular dioxygen and NAD(P)H to deformylate the Aar-produced fatty aldehyde to the corresponding C_n-1_ alkane^18,22,23^. Depending on the substrate captured by Aar, this pathway can result in the production of methyl-branched alkanes or alkenes with an internal desaturation^5^.

The second type utilizes a large, modular multi-domain enzyme called the olefin synthase (Ols) that is related to polyketide synthases^19^ (Fig. 1, red path). Ols captures fatty acyl-ACP substrates and extends the carbon chain *via* a decarboxylative condensation reaction with malonyl-CoA to generate a covalently bound C_n+2_ acyl chain. This intermediate is decarboxylated to generate the C_n+1_ alkene product with a terminal double bond, an α-olefin^19,24^. Enzymatic desaturation of acyl-ACP prior to capture by Ols results in production of doubly-desaturated 1,14-nonadecadiene^17^. The ratio of 1,14-nonadecadiene to 1-nonadecene is dramatically higher at lower growth temperatures due to increased production of singly desaturated acyl-ACP precursor^17,19^.

Genomic comparisons of cyanobacteria have shown that Ols or Ado/Aar genes are present in nearly all characterized members, though the Ado/Aar pathway is much more prevalent than Ols^5^. Interestingly, it was found during these studies that no cyanobacterium encodes genes for both of the pathways^5^. The reason for this apparent mutual exclusivity remains unknown but it could be due to a negative selection mechanism that precludes the co-existence of Ado/Aar and Ols in a cell. In order to gain insights into the compatibility between the different hydrocarbon pathways, we first studied the effects of co-expressing the Ols and Ado/Aar pathways in the model unicellular cyanobacterium *Synechococcus* sp. PCC 7002 (S7002). We then tested whether non-native hydrocarbon biosynthetic pathways could functionally substitute for Ols in a knockout strain of S7002. Our data show that Ado/Aar from other model cyanobacteria as well as disparate hydrocarbon biosynthetic enzymes from non-cyanobacteria including *Pseudomonas* and eukaryotic green algae are able to replace Ols and rescue the slow growth of alkene-deficient S7002. This suggests that these diverse pathways are interchangeable to some extent. Our results also show that the biosynthetic source of alka/enes is not critical to serving their role and various types of alka/ene hydrocarbons are able to support growth of S7002. This work implies malleability in bacterial hydrocarbon synthesis and could enable strategies for expanding the biosynthetic range of accessible alka/ene biofuels from CO_2_ using cyanobacteria.

## Results

### Introduction of the Ado/Aar genes into S7002

To express the Ado/Aar pathway in S7002, we constructed a series of autonomously replicating plasmids derived from the broad host-range vector pVZ321^25^. The Materials and Methods section provides details on plasmid assembly and Supplementary Figure S1 diagrams the construction workflow. Briefly, we first generated a derivative vector pSL3067 from the broad-host range plasmid pVZ321 that contains a kanamycin resistance cassette and the mobilization/replication genes needed for passage through *Escherichia coli* and for conjugation into S7002. Alkane expression vectors were then generated from pSL3067 using Gibson Assembly^26^. Genotypes for strains characterized in this study are presented in Supplementary Table S1 and oligos in Supplementary Table S2. To construct the initial set of vectors, we cloned the alkane biosynthetic operons from the two model cyanobacteria *Synechococcus elongatus* PCC 7942 (S7942) and *Synechocystis* sp. PCC 6803 (S6803) for introduction into S7002 *via* tri-parental mating. The empty plasmid pSL3067 was introduced into S7002 to be used as a control (strain 7002::control). The S6803 *ado* and *aar* genes were introduced into S7002 on plasmid pSL3071 to make strain 7002::6803alk. The alkane operon from S7942 containing genes *ado*, *aar*, and *accA* was initially introduced in vector pSL3070 under control of the native S7942 promoter. However, this strain did not produce detectable amounts of non-native hydrocarbons under any growth conditions. We determined that this was due to the S7942 alkane gene promoters being inactive in S7002 (Supplementary Fig. S2) and re-designed the expression vector by placing the genes under control of the synthetic promoter P_trc1O_^27^. This vector, pSL3072, was introduced into S7002 to make strain 7002::trc7942alk.

### Effects of *ado*/*aar* and *ols* co-expression on S7002 growth and hydrocarbon content

**Figure 2:**
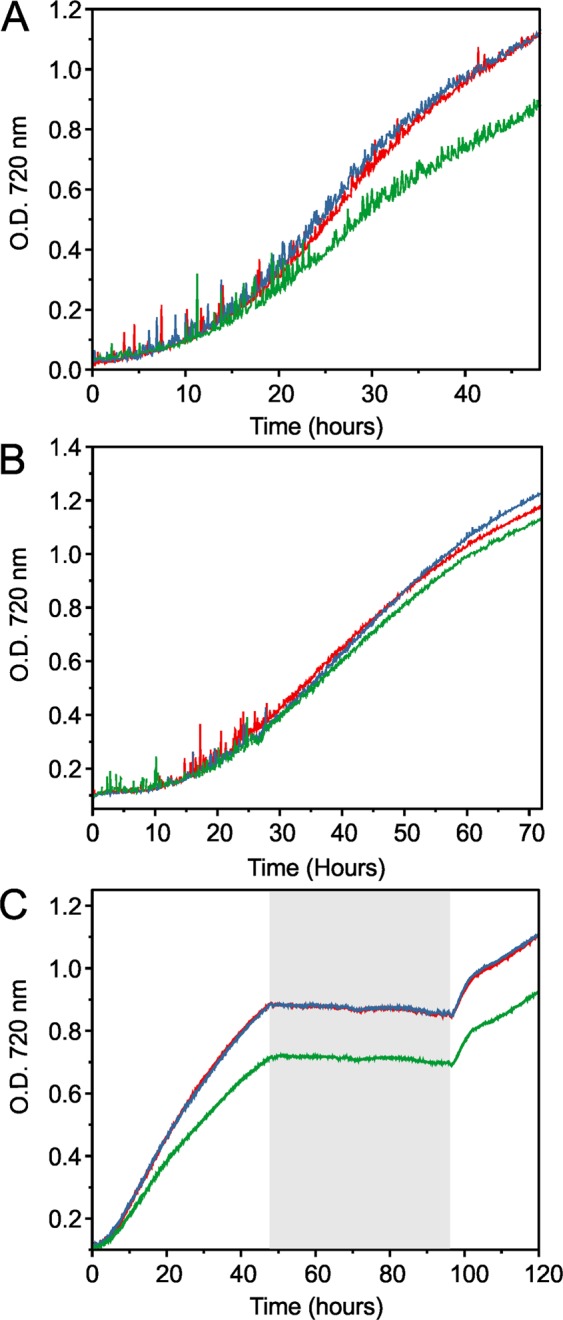
Representative growth curves of S7002 strains co-expressing Ols and the Ado/Aar pathways. In all panels, 7002::control is shown in red, 7002::6803alk in blue, and 7002::trc7942alk in green. (**A**) Strains grown at 38 °C. (**B**) Strains grown at 27 °C. (**C**) Strains exposed to a temperature and light change during growth. Strains were grown in a bioreactor under 300 μmol LED photons s^−1^m^−2^ and bubbled with air supplement with 1% CO_2_ (panels A and B) or only air (panel C). In panel C, the gray box indicates the two-day 15 °C dark period. Cells were grown in a bioreactor under 300 μmol LED photons s^−1^m^−2^ and bubbled with air supplement with 5% CO_2_.

We compared the growth of the control, 7002::6803alk and 7002::trc7942alk strains in a bioreactor at 38 °C and 27 °C while bubbling with air supplemented with 5% CO_2_ under 300 μmol LED photons s^−1^ m^−2^ illumination. We determined the maximum exponential and linear growth rates for the cultures and a wild-type (WT) S7002 as a reference (Table 1). At 38 °C, growth of 7002::control and 7002::6803alk were essentially the same but 7002::trc7942alk grew more slowly, exhibiting a longer exponential doubling time and slower linear growth rate (Fig. 2A). In contrast, growth rates of all three strains were comparable 27 °C (Fig. 2B). This was initially surprising since alka/enes had previously been shown to have a more important effect on strain growth as temperature is lowered^15,17^. Given the apparent temperature-dependent effect, we tested whether a large decrease in growth temperature and light intensity during culturing differentially affected the three strains. Each recovered from the two-day cold period at a similar rate (Fig. 2C), suggesting that Ado/Aar, even when overexpressed, do not impact S7002 temperature adaptation.

**Table 1:** Exponential and linear growth rates of S7002 strains.

Strain	Exponential doubling time in hours at 38 °C [27 °C]	Linear phase growth rate at 38 °C [27 °C]*
WT S7002	4.7 ± 0.1 [7.1 ± 0.3]^†^	0.045(2) [0.028(1)]
7002::control	6.1 ± 1 [11 ± 1.4]	0.037(2) [0.023(1)]
7002::6803alk	6.8 ± 1 [12 ± 0.7]	0.038(1) [0.023(1)]
7002::trc7942alk	7.2 ± 1 [11 ± 0.7]	0.024(4) [0.020(2)]
7002ΔOls	9.7 ± 2 [ND]^‡^	0.0190(6) [ND]
ΔOls::control	21 ± 2 [ND]	0.011(1) [ND]
ΔOls::6803alk	8.0 ± 1 [27 ± 10]	0.022(2) [0.003]
ΔOls::7942alk	7.1 ± 1 [21 ± 2]	0.029(2) [0.005]
ΔOls::UndA	7.7 ± 1 [28 ± 10]	0.023(1) [0.003]
ΔOls::FAP	7.5 ± 0.8 [16 ± 2]	0.029(2) [0.008]

*Measured in O.D. 720 nm/ hour.

^†^Errors are 1 SD based on three biological replicates.

^‡^‘ND’, not determined.

**Figure 3:**
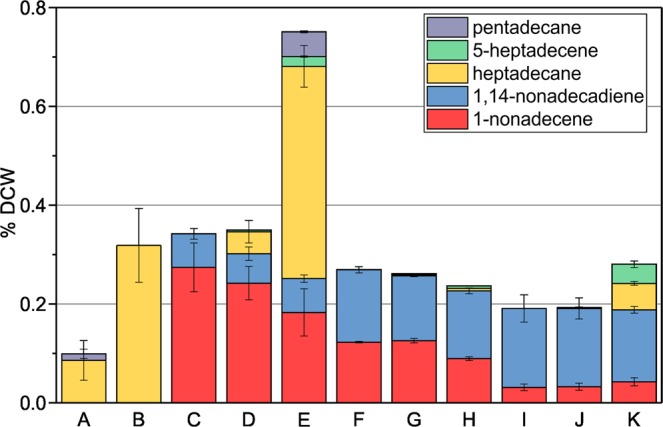
Alka/ene hydrocarbon content of the S7002 strains expressing Ols and Ado/Aar pathways and WT references. Hydrocarbon content was normalized as percent of dry cell weight (% DCW). (A) S7942 at 38 °C. (B) S6803 at 30 °C. (C) 7002::control at 38 °C. (D) 7002::6803alk at 38 °C. (E) 7002::trc7942alk at 38 °C. (F) 7002::control at 27 °C. (G) 7002::6803alk at 27 °C. (H) 7002::trc7942alk at 27 °C. (I) 7002::control at 25 °C. (J) 7002::6803alk at 25 °C. (K) 7002::trc7942alk at 25 °C. A to H were cultured under 300 μmol photons s^−1^ m^−2^ and bubbled with 5% CO_2_-supplemented air and I to K at 50 μmol photons s^−1^ m^−2^ in air. S6803 was cultured at 30 °C since the strains is not tolerant of 38 °C. Error bars are 1 SD based on at least three biological replicates.

In order to determine the extent to which Ado/Aar affect hydrocarbon pools in S7002, we quantified hydrocarbons in methanolic extracts of cells grown in the bioreactor. Methanolic extracts of dried cells were analyzed using gas chromatography (GC) and hydrocarbon content was normalized as percent dry cell weight (% DCW, Fig. 3). Hydrocarbon content of WT S7942 and WT S6803 were also determined for comparison (Fig. 3A and B). S7002 natively produces two types of α-olefins: 1-nonadecene (C_19:1_) and 1,14-nonadecadiene (C_19:2_)^5,19^. At 38 °C, the 7002::control strain produced 0.35% DCW hydrocarbons (Fig. 3C). In transitioning from 38 °C to 27 °C, the ratio of C_19:2_ to C_19:1_ increased from 0.25 to 1.2 while the overall alkene content decreased to 0.26% DCW. Co-expression of S6803 Ado/Aar in 7002::6803alk afforded production of C_17_ hydrocarbons in addition to the native α-olefins (Fig. 3D). Heptadecane was the major product but we also observed another metabolite at 38 °C that eluted shortly before heptadecane (starred in Supplementary Fig. S3). Due to the elution time and the available acyl-ACP substrates in S7002, we hypothesized that this metabolite was likely 5-heptadecene produced by action of Ado/Aar on the internally desaturated C_18:1(Δ13)_ acyl-ACP precursor of 1,14-nonadecadiene^17,19^. The mass spectrum of this molecules was consistent with a singly desaturated C_17_ hydrocarbon (Supplementary Fig. S4), either 8- or 5-heptadecene. Since S7942 naturally produces traces of 8-heptadecene^11^, we compared extracts of WT S7942 to 7002::6803alk which showed that the metabolite observed was not 8-heptadecene (Supplementary Fig. S5). At 38 °C in 7002::6803alk, cells contained predominantly native C_19_ alkenes and Ado/Aar-derived hydrocarbons amounted to only 10% of the total in the strain (Fig. 3D). The effects of Ado/Aar were further reduced at 27 °C where C_17_ hydrocarbons were barely detectable, constituting only 1.5% of alka/enes in cells (Fig. 3G). At 38 °C, 7002::trc7942alk produced a high level of non-native alka/enes, and a total hydrocarbon content of 0.75% DCW (Fig. 3E). Three hydrocarbons were detected in addition to the native alkenes: pentadecane, 5-heptadecene, and heptadecane (Supplementary Fig. S3). The amounts of the non-native alka/enes in 7002::trc7942alk exceeded the levels of native alkenes by more than two-fold and the total alkane content was also two- to three-fold that of the control strain. However, growth at 27 °C dramatically reduced production of the non-native hydrocarbons in 7002::trc7942alk to only 4% of the total (Fig. 3H). The hydrocarbon profile was close to that of the control strain, providing a plausible explanation for the reduced growth rate of 7002::trc7942alk at 38 °C but not 27 °C. We considered that the relatively high light intensity used to grow the strains at 27 °C could have affected hydrocarbon production. To test this, we cultured the three strains in shaker flasks at 25 °C in air under fluorescent lights at 50 μmol photons s^−1^ m^−2^ and quantified alka/ene content (Fig. 3I to K). The strains again grew at similar rates (Supplementary Fig. S6) but 7002::trc7942alk contained higher quantities of Ado/Aar-derived C_17_ hydrocarbons. Compared to at 27 °C in the bioreactor, heptadecane levels were increased 10-fold to 0.054% DCW and 5-heptadecene 8-fold to 0.039% DCW. Notably, these quantities were still only 18% of Ado/Aar-derived hydrocarbons produced at 38 °C. 7002::6803alk did not produce appreciable quantities of non-native hydrocarbons when grown at these conditions.

We hypothesized that the marginal effects of S6803 Ado/Aar might be due to S7002 up-regulating *ols* to maintain a near-normal level of alkenes in response to consumption of acyl-ACPs by Ado/Aar. Using quantitative RT-PCR, we determined that *ols* was expressed at similar levels in all strains and is not up-regulated even when Ado/Aar were overexpressed (Supplementary Fig. S7). Furthermore, transcript levels for the key fatty acid biosynthetic genes *accB* and *fab*^20^ were also mostly unaffected by the presence of Ado/Aar. Ols is apparently able to continue competing effectively for acyl-ACP substrates without up-regulation. We conclude from these observations that Ado/Aar and Ols can coexist in S7002 and that even overexpression of the non-native pathways is not obviously harmful to S7002.

### Substitution of S7002 Ols with Ado/Aar enzymes and effects on growth and hydrocarbon content

**Figure 4:**
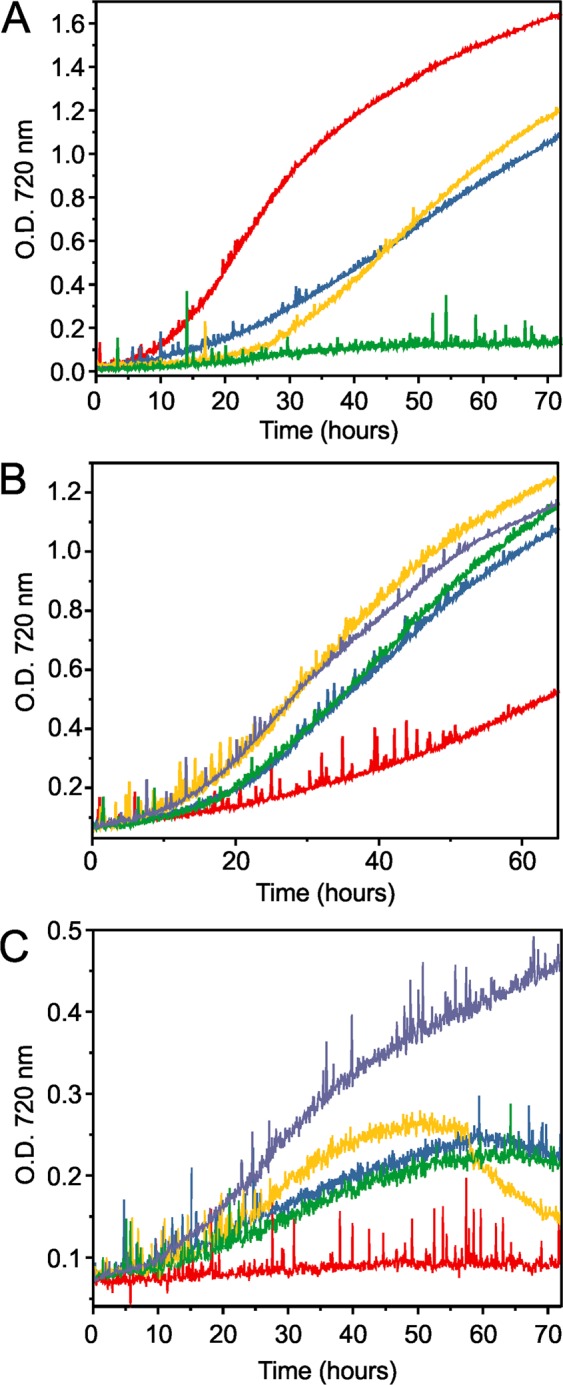
Representative growth curves of WT S7002, 7002ΔOls and Ols-replacement strains at 38 °C and 27 °C. (**A**) WT S7002 and 7002ΔOls at 38 °C (red and blue traces, respectively) and at 27 °C (yellow and green traces). (**B**) ΔOls strains grown at 38 °C. ΔOls::control (red trace), ΔOls::6803alk (blue), ΔOls::7942alk (yellow), ΔOls::UndA (green), and ΔOls::FAP (grey). (**C**) ΔOls strains grown at 27 °C, indicated using the same colors as in panel B. Strains were cultured as in Fig. 3.

Since S7002 was able to tolerate Ado/Aar, we were curious to determine the limits of hydrocarbon pathway inter-compatibility and thus asked the question of whether Ado/Aar could functionally replace Ols in a knockout strain of S7002. We knocked out olefin production in S7002 by replacing the start codon and promoter region of the Ols gene with a spectinomycin resistance cassette using natural transformation and double homologous recombination with a linear PCR fragment. This 7002ΔOls strain was fully segregated after two weeks of re-patching on selective media and no longer produced either type of C_19_ alkene (Supplementary Fig. S8). In a bioreactor, growth of 7002ΔOls at 38 °C was markedly reduced relative to WT 7002 and at 27 °C 7002ΔOls was unable to grow (Fig. 4A and Table 1). We tested whether Ado/Aar could rescue this phenotype by introducing the S6803 and S7942 Ado/Aar pathways into 7002ΔOls on plasmids. S6803 genes were expressed from pSL3071 (strain ΔOls::6803alk). The S7942 *ado, aar*, and *accA* genes were expressed from plasmid pSL3258 that uses the endogenous S7002 A2813 promoter described by Ruffing *et al*.^28^ (strain ΔOls::7942alk). We assumed that A2813 would afford more modest expression levels than trc and transcriptomic data indicated that this promoter did not significantly respond to temperature change^29^. Finally, the empty plasmid pSL3067 was introduced into 7002ΔOls and used as a control strain for comparison (ΔOls::control).

Growth of ΔOls::6803alk, ΔOls::7942alk and ΔOls::control was assessed in a multicultivator at 38 °C and 27 °C. Like 7002ΔOls, ΔOls::control grew slowly at 38 °C and no growth occurred at 27 °C (Fig. 4B). Growth rates of ΔOls::control at 38 °C were significantly reduced relative to 7002ΔOls (21 hr vs. 10 hr exponential doubling time, Table 1). We are uncertain of the exact cause of this but it could be due to the additional metabolic burden imposed by plasmid replication and antibiotic resistance. 7002::control also exhibited reduction in growth rate relative to WT S7002 but this was not as pronounced as ΔOls (Table 1). Since ΔOls was already compromised, perhaps the effect was enhanced in this background. Interestingly, the expression of Ado/Aar *in trans* rescued this phenotype and both strains grew at both temperatures (Fig. 4B). At 38 °C, exponential doubling times were greatly improved to 8 and 7.1 hrs for ΔOls::6803alk and ΔOls::7942alk, respectively, compared to 21 hours for the knockout control strain. Linear phase growth rates were similarly improved (Table 1). However, expressing Ado/Aar had only marginal positive effect on growth at 27 °C. While the strains were able to be cultured better than ΔOls::control at this temperature, they only survived for a few days before growth halted and cell density began to decrease (Fig. 4C). Growth rates at this temperature were also markedly reduced relative to the strains expressing Ols at 27 °C (compare to Fig. 2C).

**Figure 5:**
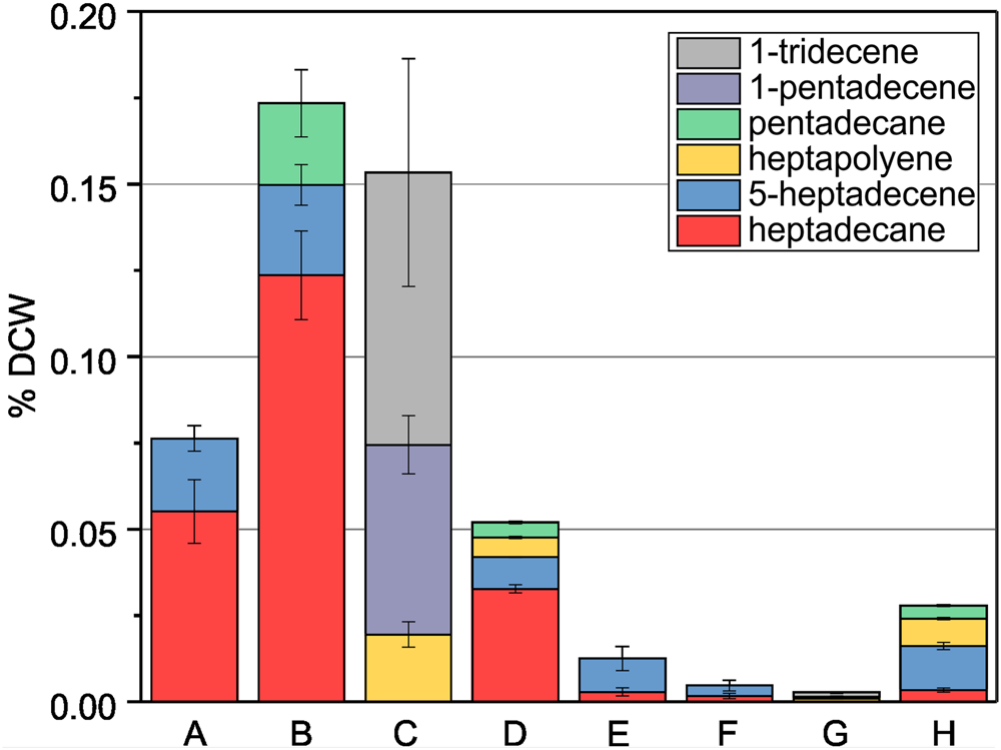
Alka/ene content of 7002ΔOls substituted with non-native hydrocarbon biosynthetic genes presented as % DCW. (A) ΔOls::6803alk at 38 °C. (B) ΔOls::7942alk at 38 °C. (C) ΔOls::UndA at 38 °C. (D) ΔOls::FAP at 38 °C. (E) ΔOls::6803alk at 27 °C. (F) ΔOls::7942alk at 27 °C. (G) ΔOls::UndA at 27 °C. (H) ΔOls::FAP at 27 °C. Strains were cultured as in Fig. 3. Error bars are 1 SD based on at least three biological replicates.

We quantified alkane production in these Ado/Aar-replacement strains. ΔOls::6803alk produced exclusively C_17_ hydrocarbons, mostly heptadecane and some 5-heptadecene (Fig. 5A and Supplementary Fig. S9). At 38 °C, these hydrocarbons amounted to 0.075% DCW. Similarly, ΔOls::7942alk produced 0.17% DCW alka/enes at 38 °C consisting of heptadecane, 5-heptadecene, and pentadecane, in decreasing amounts (Fig. 5B and Supplementary Fig. S9). In contrast, when grown at 27 °C, these strains produced very few alka/enes. In ΔOls::6803alk, total hydrocarbons were reduced to 0.012% DCW and in ΔOls::7942alk to only 0.005% DCW (Fig. 5E and F). This corresponds to an 84% reduction in alka/enes content for ΔOls::6803alk and >97% in ΔOls::7942alk. We considered that decreased expression of the *ado* and *aar* genes at 27 °C might be responsible and performed semi-quantitative RT-PCR on ΔOls::6803alk and ΔOls::7942alk. However, the results indicated that the expression levels were not significantly impacted by the temperature change in either strain (Fig. S10).

### Replacement of Ols with non-cyanobacterial alka/ene-producing enzymes UndA and fatty acid photodecarboxylase

Given the success of replacing Ols with Ado/Aar at 38 °C, we asked whether non-cyanobacterial alka/ene-producing pathways would be able to similarly substitute for Ols and recover the slow-growth phenotype of ΔOls. This experimental approach also afforded us the opportunity to find enzymes that might not exhibit the temperature-dependent effects displayed by Ado/Aar. To test this, we chose two other alka/ene-synthesizing enzymes, UndA from *Pseudomonas aeruginosa*^30^ and the fatty acid photodecarboxylase (FAP) from green algae^31^ to test in the 7002ΔOls background. FAP catalyzes the blue light-driven synthesis of alkanes from fatty acids *via* decarboxylation^31^. The non-heme iron enzyme UndA and its homologues catalyze the oxidative decarboxylation of fatty acids to produce terminal alkenes in *Pseudomonas* species^30^. The UndA gene from *Pseudomonas aeruginosa* PA14 (PA14_53120) was synthesized and placed under control of the A2813 promoter on a vector (pSL3208). The FAP gene from *Chlorella variabilis* NC64A (GenBank accession KY511411) was synthesized and cloned into an expression vector under control of the A2813 promoter (pSL3244).

**Figure 6:**
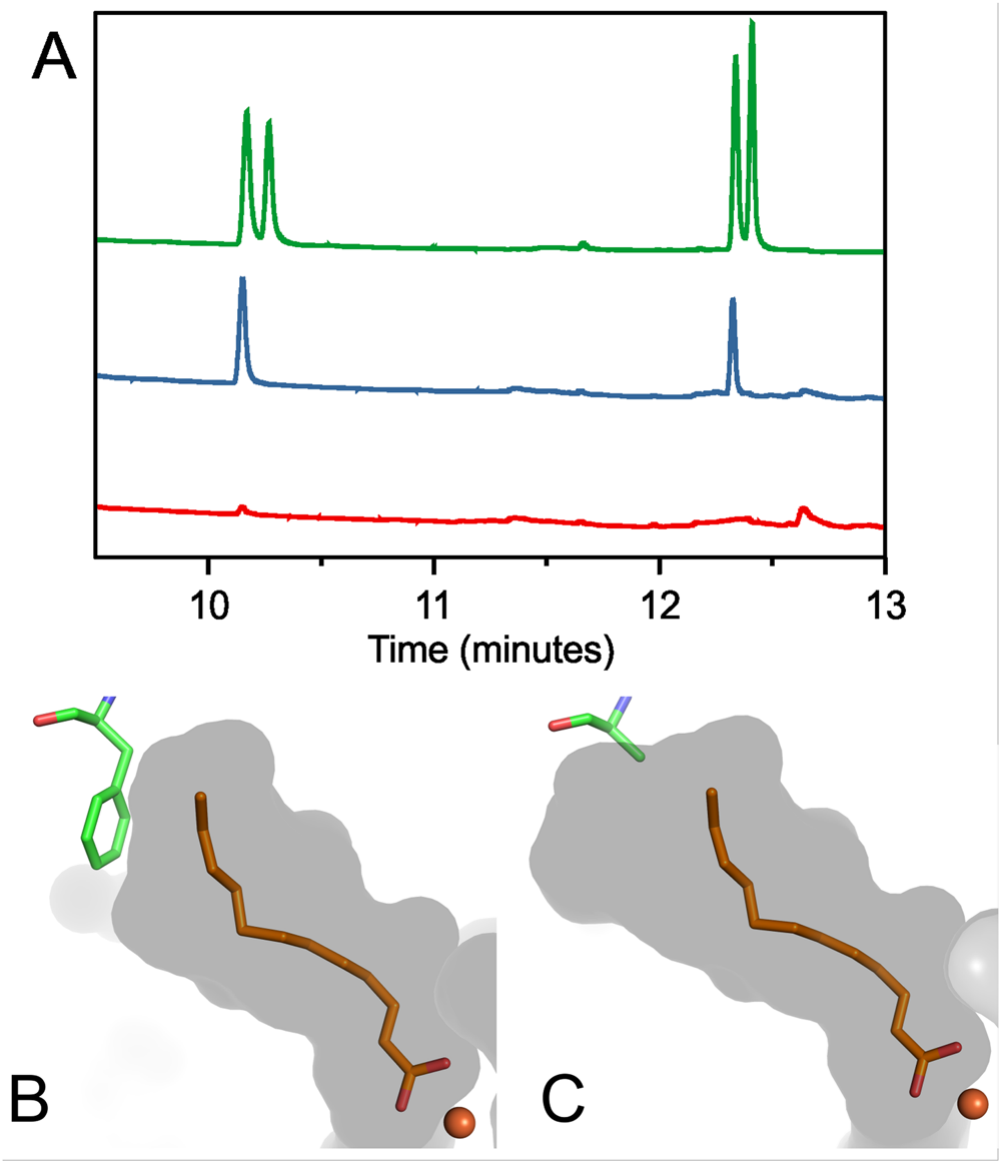
Mutation of *Pseudomonas aeruginosa* UndA to increase substrate range. (**A**) GC traces of S7002 cell extracts expressing native UndA (red trace) and the UndA-F239A mutant (blue). The green trace shows hydrocarbon standards for C_13:1_, C_13_, C_15:1_, and C_15_ in order of elution. (**B**) Substrate pocket in WT UndA. (**C**) Predicted expanded substrate pocket in UndA F239A. In B and C, the substrate pocket is shown as a grey surface, 2,3-dodecenoic acid substrate in orange and the iron ion as a brown sphere. UndA structure images were made using Pymol version 2.0.7, Schrödinger, LLC. Sample inputs for GC in panel A were normalized to dry cell biomass.

We initially tested UndA for activity in WT S7002. Expression of UndA afforded minimal production of non-native hydrocarbons and only traces of 1-tridecene were detected (Fig. 6A, red trace). We hypothesized that the low activity of UndA was due to the limited substrate range of the native enzyme and its preference for mid-chain fatty acids (C_10_–C_14_) which were present in too-low amounts in S7002^30^. To increase the substrate range of UndA, we generated a single mutation based on the crystal structure of the *Pseudomonas fluorescens* Pf-5 homologue (PDB ID 4WWZ, Fig. 6B). We predicted that a mutation of phenylalanine 239 to alanine would enlarge the substrate pocket (Fig. 6C) and allow reactions with longer substrates such as hexadecanoic acid, which is the major fatty acid in S7002^32^. This rational enzyme engineering approach was successful and UndA-F239A produced 1-pentadecene and increased quantities of 1-tridecene in WT S7002 (Fig. 6A, blue trace). Detection of 1-pentadecene indicates a reaction of the mutant enzyme with hexadecanoic acid substrate. The increased production of 1-tridecene also suggests that the activity towards tetradecane was improved by the mutation. UndA-F239A was introduced *in trans* into 7002ΔOls on plasmid pSL3209 to make strain ΔOls::UndA.

### Growth rates and hydrocarbon content of UndA- and FAP-substituted 7002Δols

Both UndA and FAP were able to support growth of 7002ΔOls at 38 °C resulting in similarly improved doubling times as the Ado/Aar-substituted strains (Fig. 4B and Table 1). However, at 27 °C, we observed a differential response of the two strains. ΔOls::UndA performed poorly and growth ceased after about two days, similar to ΔOls::6803alk and ΔOls::7942alk (Fig. 4C). In contrast, ΔOls::FAP growth was markedly improved compared to the other strains (Fig. 4C), doubling in 16 hours compared to >21 hours for the others. The strain also did not cease growth after a few days but did reach a lower culture density than strains with Ols.

**Figure 7:**
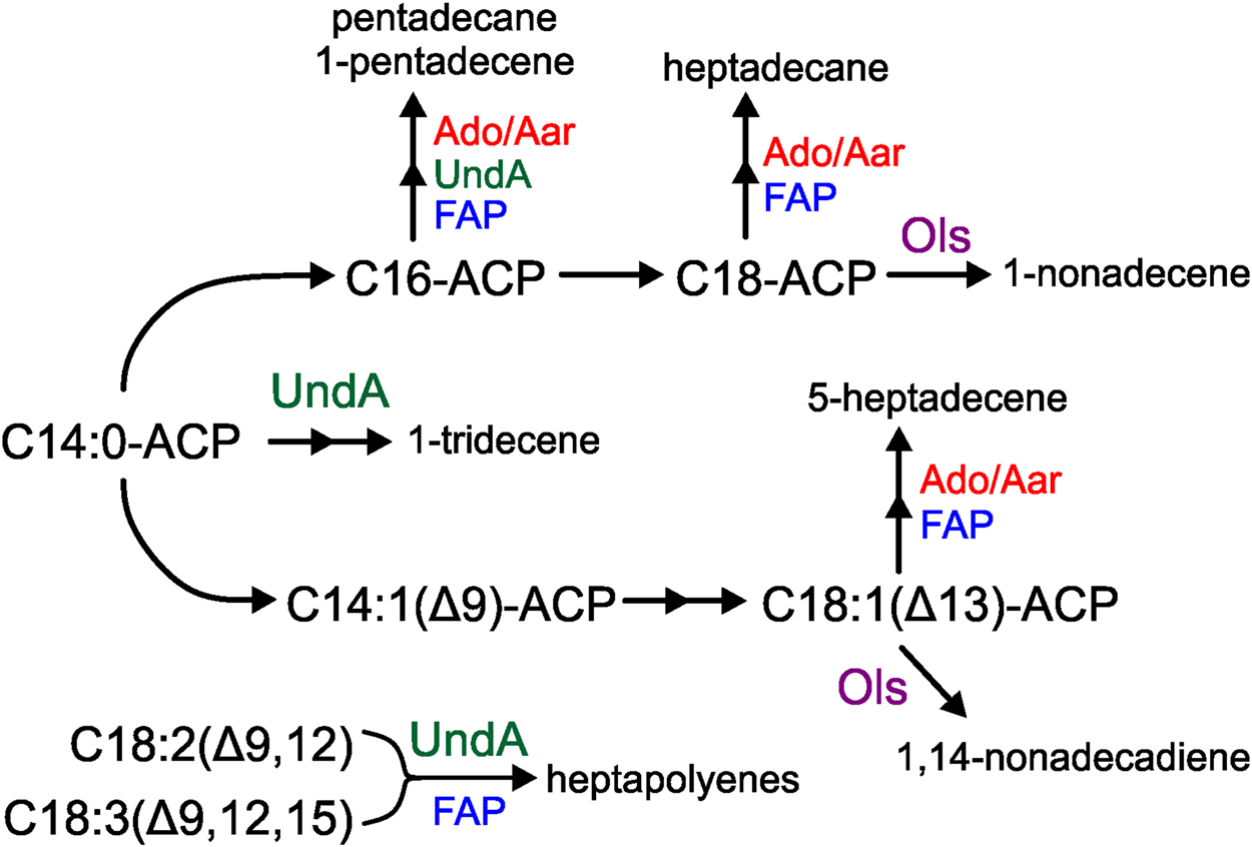
Summary of substrates and hydrocarbon products generated by Ols and non-native pathways in S7002. The substrates for Ado/Aar and Ols are acyl-ACPs and those for UndA and FAP are the equivalent fatty acids likely derived from turnover of membrane lipids.

We quantified alka/ene content in ΔOls::UndA and ΔOls::FAP under the two conditions in order to determine why FAP afforded improved growth at 27 °C relative to the other strains. ΔOls::UndA produced mainly the shorter alkenes 1-tridecene and 1-pentadecene (Fig. 5C and Supplementary Fig. S9). ΔOls::FAP produced primarily heptadecane, 5-heptadecene and pentadecane resulting in a hydrocarbon profile similar to that of ΔOls::7942alk but with a lower overall content (Fig. 5D, and Supplementary Fig. S9). In both strains, we observed metabolites in the GC traces that were not seen in any of the other strains (starred in Supplementary Fig. S9). The elution time suggested that these were C_17_ compounds and we hypothesized that they were alkenes derived from reaction of UndA F239A with linoleic (C_18:2(Δ9,12)_) and α-linolenic (C_18:3(Δ9,12,15)_) fatty acids present in S7002^32^. Feeding of these fatty acids to ΔOls::UndA cultures resulted in increased production of the two metabolites (Supplementary Fig. S11), confirming our hypothesis and identifying these compounds as heptapolyenes containing two to four desaturation sites. ΔOls::UndA also contained traces of heptadecane, but these were too low to accurately quantify. The production of C_17_ alkenes by UndA F239A suggests that the mutation successfully extended the substrate range from C_14_ up to C_18_ fatty acids, which has not been previously shown. At 38 °C, ΔOls::UndA contained 0.15% DCW hydrocarbons and ΔOls::FAP contained 0.05% DCW (Fig. 5C and D). Although ΔOls::FAP produced the lowest total alka/ene content of the replacement strains at 38 °C (less than one third that of ΔOls::7942alk), the strain was still among the best-performing. At 27 °C, ΔOls::UndA hydrocarbons were reduced >98% whereas ΔOls::FAP maintained 0.028% DCW alka/enes corresponding to only a 54% reduction relative to 38 °C (Fig. 5G and H). In ΔOls::FAP, the decrease in hydrocarbons was mainly due to a major reduction of heptadecane production. Content of the other hydrocarbons 5-heptadecene and heptapolyenes was slightly increased. Of the four strains tested, ΔOls::FAP maintained the highest level of hydrocarbons upon shifting to 27 °C, providing a likely explanation for its superior growth relative to the other Ols-substituted strains. The various products generated by the non-native pathways in S7002 and their substrates are summarized in Fig. 7.

## Discussion

The ability of disparate hydrocarbon biosynthetic pathways from bacteria and eukaryotes to conditionally substitute for Ols in S7002 has several important implications. The effect of the non-native pathways on S7002 growth rates and hydrocarbon pools and growth rate is critically dependent on growth temperature. With the exception of FAP, growth at 27 °C dramatically reduced the production of alka/enes by the non-native pathways in S7002 by 84–98%. It is likely that there is a threshold quantity of hydrocarbons needed per cell in order to facilitate growth and the low quantities produced by most of the ΔOls strains at 27 °C are insufficient to support growth. There are several plausible explanations for the reduction in alka/ene production. The first is that Ado/Aar preferentially react with saturated acyl-ACP substrates that are in increased supply at 38 °C. This preference is evidenced, in part, by the ratio of 5-heptadecene to heptadecane (0.07, 0.05) vs. the ratio of 1,14-nonadecadiene to 1-nonadecene (0.24, 0.37) produced in 7002::6803alk and 7002::trc7942alk at 38 °C. Due to this preference, the increased desaturation of acyl-ACPs in S7002 at lower temperature reduced the impact of Ado/Aar due to a shift to less favorable desaturated substrates. This essentially has the effect sequestering a pool of desaturated acyl-ACPs for reaction with Ols and not Ado/Aar. The production of fatty acids for UndA could be similarly affected by the increased acyl-ACP desaturation. We observed mainly production of 1-tridecene and 1-pentadecene in ΔOls::UndA, suggesting that the enzyme is reactive towards tetradecanoic acid and hexadecanoic acid and not their internally desaturated analogues which would have resulted in production of alkadienes. Since myristic acid is the major substrate for UndA at 38 °C, increased desaturation of C_14_-ACP to C_14:1(Δ9)_-ACP at 27 °C would reduce production of the corresponding saturated fatty acids and thereby limit production of UndA-derived alkenes. In contrast, FAP is able to produce predominantly 5-heptadecene at 27 °C by reaction with C_18:1(Δ9)_ fatty acid, giving it an advantage over UndA since it is more compatible with the internally desaturated substrates.

The second reason for the reduction may be tied to a differential availability of reducing equivalents for the enzymes at 27 °C vs. 38 °C. Previous reports show that S7002 still contains quantities of palmitic and stearic acid at 22 °C^33^ which would be viable substrates for FAP and, if activated to acyl-ACPs, for Ado/Aar. The delivery of reducing equivalents to Ado and/or Aar could be compromised at the lower temperature due to photoinhibition and/or redox imbalance at 27 °C. We see some evidence for this. At 27 °C, production of 1-nonadecene in 7002::trc7942alk is reduced relative to the control, possibly due to capture by Aar. However, there is little production of Ado/Aar -derived alka/enes (Fig. 3H). If electron delivery to Ado is compromised at 27 °C, the coupling between Aar acyl-ACP reduction and Ado decarbonylation would result in incomplete turnover of acyl-ACPs into alka/enes. Furthermore, when 7002::trc7942alk was cultured under low light conditions in shake flasks at 25 °C, it contained eight- to ten-fold more non-native hydrocarbons. The decreased light intensity and overall slower growth rate of the strains could have resulted in less photoinhibition and higher Ado/Aar activity. While the electron-donating co-substrate for the UndA reaction is still in question^30^, a similar inefficient delivery of reducing equivalents to the enzyme at 27 °C could contribute to its low activity at this temperature. Interestingly, the use of light as a substrate in the decarboxylation reaction by FAP obviates the need for electron delivery by ferredoxin or other redox partners^31^. This could provide another explanation for this enzyme’s better relative performance at 27 °C.

The story is quite different at the higher growth temperature. The improved growth exhibited by the Ols-replacement strains compared to the knockout indicates that these disparate biosynthetic pathways are able to support healthy growth of S7002 at 38 °C. This observation has several implications. First, the biological function of hydrocarbons is not necessarily tied into the specific structure of the alka/ene molecules. Expression of UndA and FAP in S7002 afforded unique hydrocarbon profiles not observed in any other wild cyanobacteria^5^. Hydrocarbons with various chain lengths from C_13_ to C_17_ containing different desaturation profiles are able to serve the same role as the native C_19_ α-olefins in S7002. Secondly, it means the producing enzyme and the source of the substrates are not critical. The biosynthetic mechanisms of these enzymes are distinct, as are their preferred substrates. The Ols and Ado/Aar enzymes use acyl-ACP substrates whereas UndA and FAP react with fatty acids. Since free fatty acids are activated to acyl-ACPs in cyanobacteria, the source for the UndA and FAP substrates is likely to derive from turnover of membrane lipids^34^. Capture and consumption of these molecules apparently does not greatly impact growth of S7002 compared to Ado/Aar and both types of enzymes are able to support growth of S7002 at 38 °C.

One of prevailing hypotheses for the role of alka/enes is that they partition into the interior of membrane bilayers and thereby afford cells a means to modulate membrane fluidity and structure in response to a variety of stimuli including light and temperature changes, during cell division, and fluctuating thylakoid lumen pressure resulting from proton pumping^14,15,35–37^. The ability of different types of hydrocarbons to support growth of S7002 in consistent with the hypothesis that these molecules primarily serve basal structural roles in cells. Although different kinds of hydrocarbons can serve this role, regulated and steady production of alka/enes in response to changing growth conditions is critical. In a less controlled growth environment, the native Ols pathway is likely to afford an advantage over Ado/Aar, UndA, and FAP for S7002 since it is able to continue producing hydrocarbons in response to changing temperature. This provides a plausible explanation for why Ols is maintained over Ado/Aar in S7002 and that enzyme compatibility with endogenous acyl-ACP and fatty acid profiles may be an important determinant of which pathway is preferred in a cyanobacterial cell.

In conclusion, we found that Ado/Aar and Ols pathways are not mutually exclusive and S7002 is able to support the co-existence of both pathways. Far from being incompatible, alka/ene biosynthetic pathways from disparate organisms and domains of life are able to support growth of S7002. While the native Ols pathway in S7002 does display an advantage over the exogenous pathways, we hypothesize that this has more to do with a compatibility of Ols with the native acyl-ACP substrates over a variety of growth conditions rather than a strict requirement of α-olefin production in cells. These data suggest that the specific biosynthetic mechanism and enzyme pathway for hydrocarbon production is not critical as long as sufficient hydrocarbon production is maintained in response to changing environmental conditions. The unique hydrocarbon profiles of ΔOls::UndA and ΔOls::FAP evidence an opportunity to expand the hydrocarbon production range of cyanobacteria to chain lengths not accessed by WT strains. Our experiments only tested a subset of the hydrocarbon biosynthetic pathways found in nature^38,39^, and it will be interesting to determine to what extent these diverse pathways are fundamentally interchangeable *in vivo*.

## Materials and Methods

### Assembly of expression vectors

A diagram illustrating vector construction is shown in Supplementary Figure S1. Vector pSL3067 was constructed by sequential digestion of the broad host-range vector pVZ321^25^ with the restriction enzymes XbaI and PstI. The doubly digested fragment was ligated with a linker molecule composed of the annealed oligos linker1 and linker2 using T4 DNA Ligase (Thermo Scientific). pSL3067 contains the *aphA* gene conferring kanamycin resistance and RSF1010-derived replication, mobilization genes and ori sites^25^. pSL3067 was cut with XbaI, purified and used in Gibson Assembly to generate the expression vectors. Alkane genes were PCR-amplified from S7942 and S6803 genomic DNA (gDNA). To construct pSL3070, the *ado*-*aar-accA* genes were amplified from S7942 gDNA using primers alk1/alk2 and combined with XbaI-linearized pSL3067. The fragments were joined using Gibson assembly and the product transformed into XL1-Blue *Escherichia coli*. Transformants were selected on LB media with 50 μg/ml kanamycin. Resulting colonies were PCR screened for the correctly assembled plasmid and positive colonies cultured overnight in liquid LB with 50 μg/ml kanamycin. Plasmids were purified using a GeneJet Plasmid Miniprep Kit (Thermo Fisher Scientific) and sequences verified using Sanger Sequencing (Genewiz, www.genewiz.com). pSL3071 was constructed by amplifying *ado-aar* and their native promoters from S6803 gDNA using primers alk3/alk4 and combining the PCR fragment with XbaI-digested pSL3067 for Gibson assembly and transformation. For pSL3072, the P_trc10_ sequence was amplified from vector template pSL2801^40^ using primers alk5/alk6 and *ado*-*aar-accA* genes from S7942 gDNA using primers alk7/alk2. The fragments were Gibson assembled, transformed and isolated as described for pSL3070 and pSL3071 above. The UndA and FAP gene coding sequences were synthesized by Genewiz. pSL3208 containing the UndA coding sequence was generated using primers alk8/alk9 to amplify the A2813 promoter from S7002 gDNA and alk10/alk11 to amplify UndA from the synthesized DNA fragment. The UndA F239A point mutation was generated by amplifying two fragments from pSL3208 using the error-introducing primer pairs alk8/alk12 and alk13/alk11 followed by Gibson assembly and isolation as described for pSL3070. pSL3244 containing the FAP coding sequence was made using primer pairs alk8/alk14 to amplify the A2813 promoter sequence and alk15/alk16 to amplify FAP from the synthesized DNA fragment and with terminal overlaps for Gibson Assembly. The fragments were mixed with XbaI-cut pSL3067 and assembled and isolated as described above. Vector pSL3258 was generated by first double-digesting vector pSL3070 with KpnI and BamHI. The A2813 promoter sequence was amplified from S7002 gDNA using primers alk17/alk18 and mixed with linearized pSL3070 for assembly. To make the *ols* knockout PCR fragment, the homology regions were amplified from S7002 gDNA using primers olsUS-F/olsUS-R and olsDS-F/olsDS-R and the spectinomycin cassette from vector pAM1303 (Addgene) using primers spec-F/spec-R. These three fragments were joined using overlap extension PCR and transformed into S7002 as described^28^. To generate vector pSL3068, we digested pSL3067 with XbaI enzyme. The *eYFP* gene was amplified from vector pSL2801^40^ using primers eYFP-F/eYFP-R and used in Gibson assembly with the XbaI-linearized pSL3067. The forward primer introduces an AarI restriction site upstream of eYFP. Promoter sequences were amplified using the following primer pairs and inserted into AarI-digested pSL3068 using Gibson assembly: P_ado_, Pado-F/Pado-R; P_alk_, Palk-F/Palk-R; P_trc1O_, Ptrc1O-F/Ptrc1O-R. Cells were grown under 500 μmol LED photons s^−1^m^−2^ and 1% CO_2_-enriched air at 38 °C overnight.

### Cyanobacterial genetic manipulation and culturing conditions

S7002 was cultured in medium A supplemented with 4 μg/L Vitamin B_12_. Antibiotic concentrations in the media were 60–100 μg/ml for kanamycin and/or 40–80 μg/ml for spectinomycin. Vectors were transferred into S7002 by conjugation using tri-parental mating as described previously^41^. Optical density of cells at 730 nm was determined in 96-well culture trays using 150 μl cells and measured using a BioTek μQuant plate reader. For bioreactor growth, S7002 was cultured in a Multi-Cultivator MC1000 instrument (Photon Systems Instruments). Cells were grown in 50 mL volume in 200 × 30 mm Pyrex test tubes. Strains were acclimated to growth in the multicultivator for a few days at the requisite temperature and then re-diluted to start the experiment. The *ols* knockout was generated by natural transformation of S7002 using previously reported methods^28^. Exponential doubling times were determined from fitting the exponential growth phase using equations y = Ae^kt^ and t_1/2_ = ln(2)/k where t_1/2_ is the doubling time. Linear growth rates were calculated directly from the linear phase after exponential growth.

### Metabolite extraction and GC/MS analysis

S7002 cells were grown to O.D. 730 nm of 0.8–1.2 and harvested by centrifugation. The pelleted cells were washed with water twice, re-pelleted and frozen in liquid N_2_. Frozen cells were lyophilized overnight and weighed to determine dry biomass. Alka/enes were extracted in 100% methanol spiked with an internal hexadecane standard (at 77 μg/ml). The cell debris was pelleted and the supernatant transferred to a glass vial for analysis. Alka/enes were quantified using a HP/Agilent 5890 N gas chromatography instrument equipped with a 12 m DB5-MS column and run using the following method: 80 °C for 5 mins, ramp to 200 °C at 15 °C/min, hold for 4 mins, then ramp to 300 °C at 33 °C/min and hold for 3 mins. We injected 6–8 μl of extract for each run. Helium was used as the carrier gas. Alka/enes were identified by comparison to authentic standards (Sigma-Aldrich) when available and/or using GC/MS analysis. Metabolites were quantified from a standard curve of authentic alka/enes and normalized to an internal hexadecane standard. Concentrations of 1,14-nonadecadience and 5-heptadecene were calculated using the standard curves for 1-nonadecene and heptadecane, respectively. GC/MS analysis was performed at the Washington University Biomedical Mass Spectrometry Resource on an Agilent 7890 GC instrument running in positive electrospray ionization mode. The LC/MS data was analyzed using Agilent Mass Hunter Software.

## Supplementary information

Supplementary Information
