# Supplementary material for: Diverse hydrocarbon biosynthetic enzymes can substitute for olefin synthase in the cyanobacterium Synechococcus sp. PCC 7002
Source: Sci Rep. 2019 Feb 4;9:1360. doi: 10.1038/s41598-018-38124-y (PMC6361979; doi:10.1038/s41598-018-38124-y)
Supplement: Supplementary file 1 — Supplementary Information [file 41598_2018_38124_MOESM1_ESM.pdf]

Supplementary Information for:

Diverse hydrocarbon biosynthetic enzymes can substitute for olefin synthase in the cyanobacterium *Synechococcus sp.* PCC 7002

Cory J. Knoot and Himadri B. Pakrasi

Department of Biology, Washington University, St. Louis, Missouri 63130

Correspondence should be addressed to H.B.P (email: pakrasi@wustl.edu)

Supplementary Materials and Methods

Measurement of strain fluorescence. eYFP fluorescence was measured using a BioTek Synergy Mx plate reader in 96-well tray format. The O.D. 730 nm was measured followed by fluorescence. Cells were excited at 514 nm and fluorescence measured at 527 nm. Fluorescence was corrected for background, normalized to O.D. 730 nm and this ratio normalized to that of the control strains harboring pSL3068, which has the eYFP gene without a promoter.

RNA extraction and RT-PCR. To test expression of *ols, fabH,* and *accB*, total RNA was extracted from actively growing S7002 cells (O.D. 730 nm near 0.9) cultured at 38 °C in air. We collected RNA from three biological replicates for each strain. RNA was isolated using the RNAwiz kit (Ambion/Thermo Fischer Scientific) and DNAse-treated to remove trace DNA. We confirmed that this RNA was free of contaminating genomic DNA using PCR. cDNA was synthesized using a Superscript II Reverse Transcriptase kit (Thermo Fischer Scientific). Quantitative PCR (qPCR) was performed using the SYBR green PCR kit (Applied Biosystems) on an Applied Biosystems 7500 Real Time PCR System and quantified using the $2^{-\Delta\Delta C_T}$ method [1]. Transcript levels were internally normalized to *petB* (cytochrome b6f) RNA and are reported relative to the expression levels in 7002::control. Transcripts were amplified using the following primers: *petB*, petB-qPCR1/petB-qPCR2; *ols*, ols-qPCR1/ols-qPCR; *accB*, accB-qPCR1/accB-qPCR2; and *fabH*, fabH-qPCR1/fabH-qPCR2. For RT-PCR on strains ΔOls::6803alk and ΔOls::7942alk, cells were cultured in the Multicultivator at 38 °C or 27 °C under 300

μmol photons $s^{-1}m^{-2}$ while bubbling with air supplemented with 3% CO_2. Cells were harvested, frozen and the RNA extracted and cDNA synthesized as described above. Primers used to amplify the genes were as follows: S6803 *ado*, 6803ado-qPCR1/6803ado-qPCR2; S6803 *aar*, 6803aar-qPCR1/6803aar-qPCR2; S7942 *ado*, 7942ado-qPCR1/7942ado-qPCR2. *petB* primers were the same as above. Primers were designed to amplify short 100-120 bp products near the start codon of the genes. Since the S7942 genes are expressed from a single promoter as an operon we only assessed expression of *ado* in strain ∆Ols::7942alk. The cDNA was confirmed to be free of contaminating plasmid and genomic DNA by performing RT-PCR on the DNAse-treated RNA using the same primer pairs.

Supplementary Figures

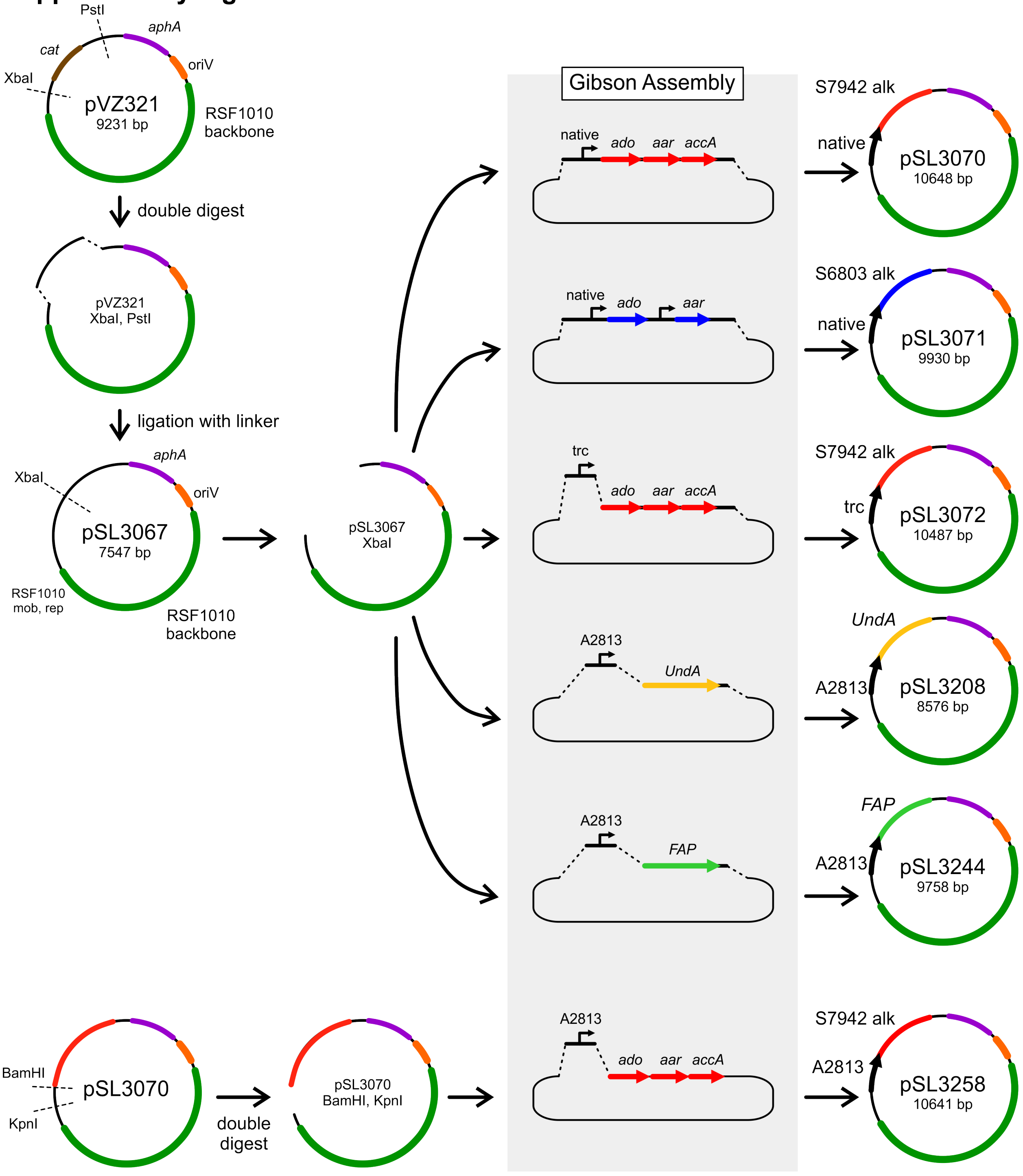

Figure S1. Workflow for assembling the plasmids used in this study. DNA fragments used in Gibson Assembly were amplified using primer pairs and templates described in the main text Materials and Methods and listed in Table S2. Maps are not to scale.

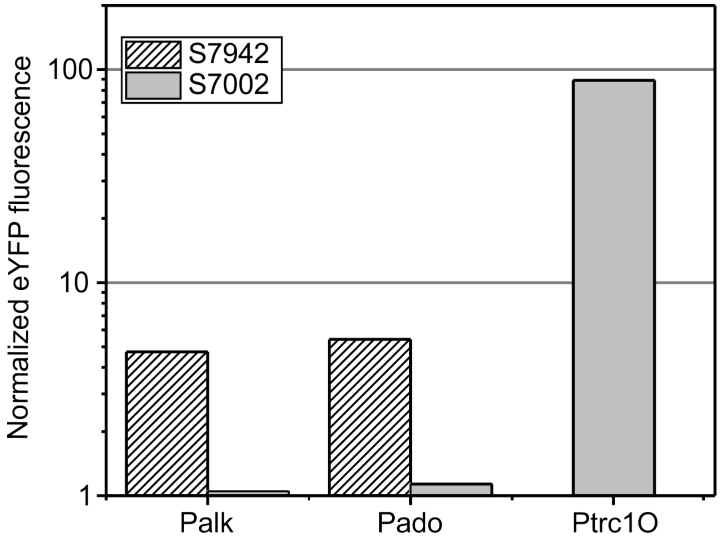

Figure S2. Fluorescence measurements of S7002 and S7942 expressing eYFP under control of the alkane gene promoters from S7942. P_{alk} and P_{ado} contain 1000 and 250 bp upstream of the S7942 *ado* gene start codon, respectively. Fluorescence was measured in S7942 and S7002 at 38 °C during the linear growth phase and the data were normalized to a control strain harboring eYFP without a promoter sequence.

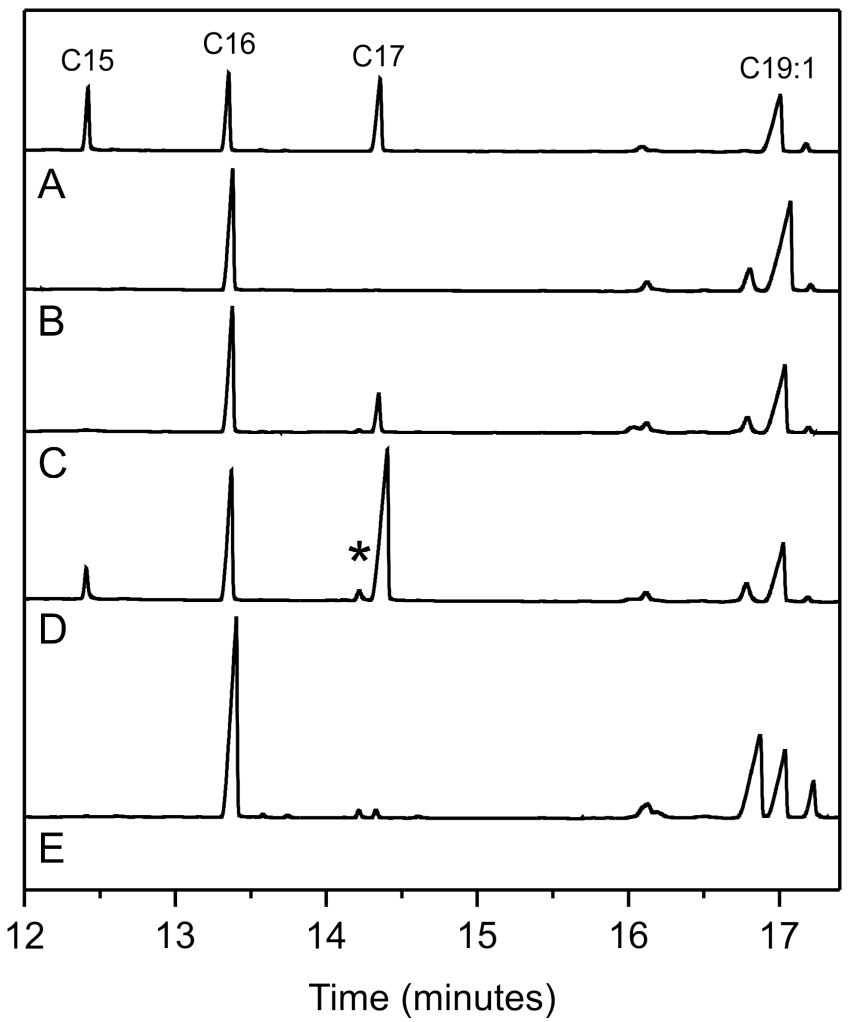

Figure S3. Gas chromatograms of methanolic extracts of S7002 strains expressing OIs and Ado/Aar pathways. (A) Authentic hydrocarbon standards. (B) 7002::control at 38 °C. (C) 7002::6803alk at 38 °C. (D) 7002::trc7942alk at 38 °C. (E) 7002::trc7942alk at 27 °C. Hexadecane is an internal standard.

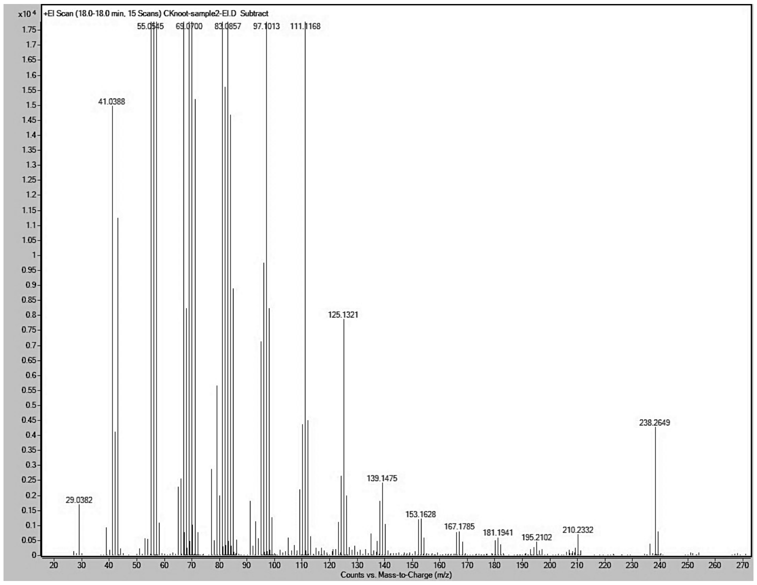

Figure S4. Mass spectrum of the analyte assigned as 5-heptadecene. The expected monoisotopic parent peak ion for 5-heptadecene is 238.26 m/z and the observed fragmentation pattern is consistent with sequential loss of methylene units.

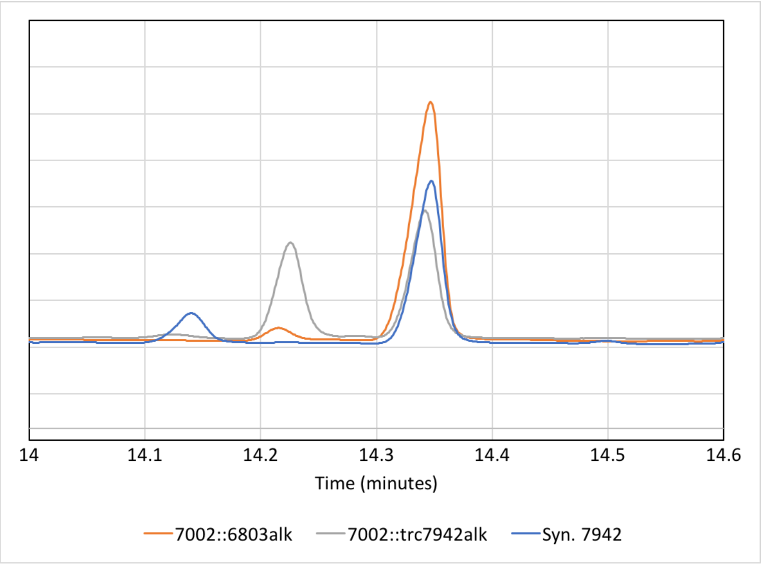

Figure S5. Comparison of GC traces for S7942 and S7002 methanolic cell extracts. Data shown are for S7942 and 7002::6803alk grown at 38 °C and 7002::trc7942alk grown at 25 °C.

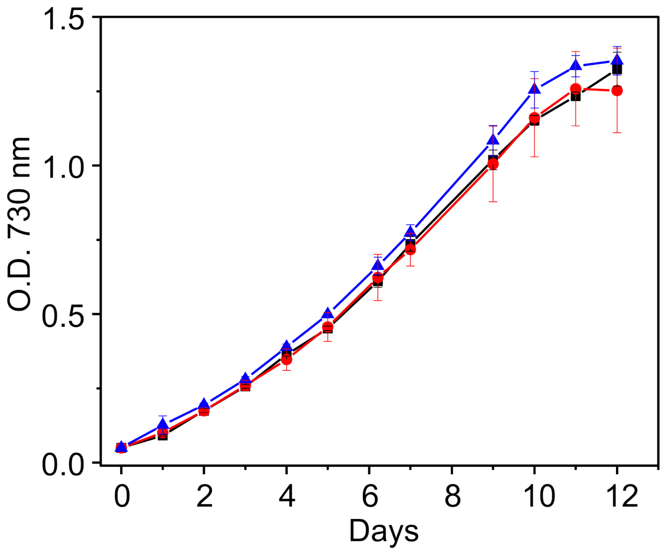

Figure S6. Growth rates of S7002 strains cultured in shake flasks at 25 °C in ambient air under 50 μmol photons $s^{-1}m^{-2}$ light intensity (fluorescent bulbs). Black: 7002::control. Red: 7002::6803alk. Blue: 7002::trc7942alk. Culture density was measured by using a plate reader. Data points are based on three biological replicates and errors are 1 SD.

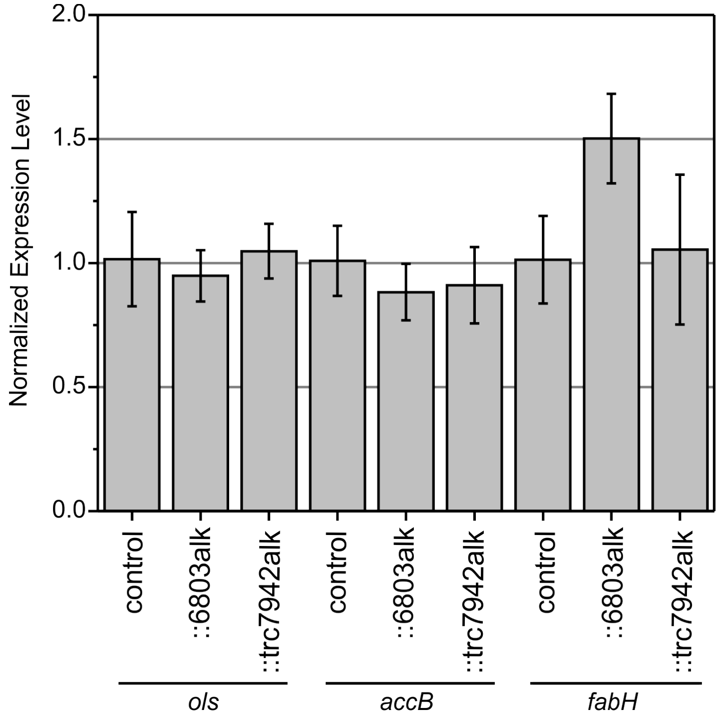

Figure S7. Comparison of *ols*, *accB* and *fabH* gene expression levels in S7002 strains using quantitative RT-PCR of strains grown at 38 °C. Data are based on three biological replicates each. Expression levels were normalized to *petB*. Errors are 1 SD.

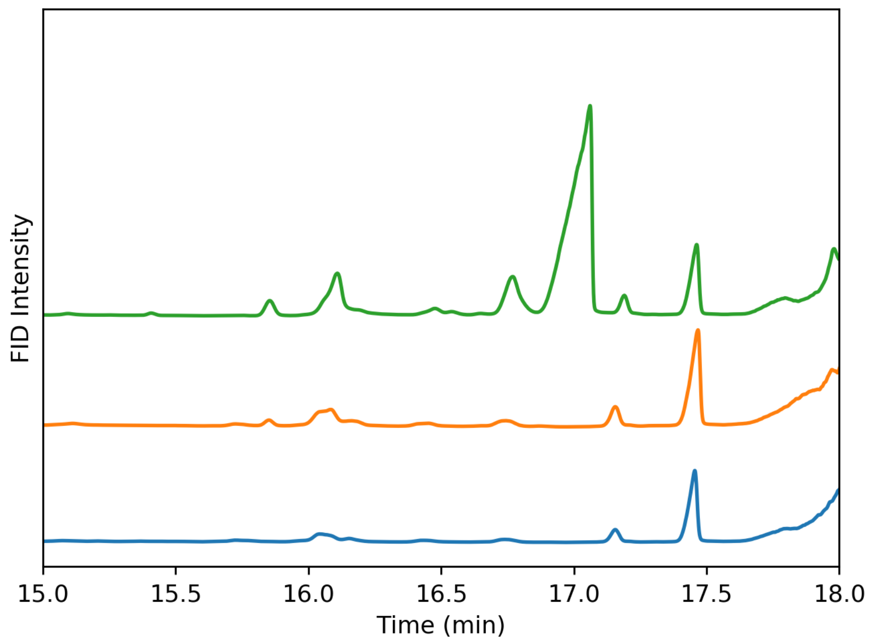

Figure S8. Gas chromatograms of WT S7002 (green) and 7002ΔOls (orange) organic extracts and methanol solvent (blue). 7002ΔOls does not produce either type of C_{19} alkene.

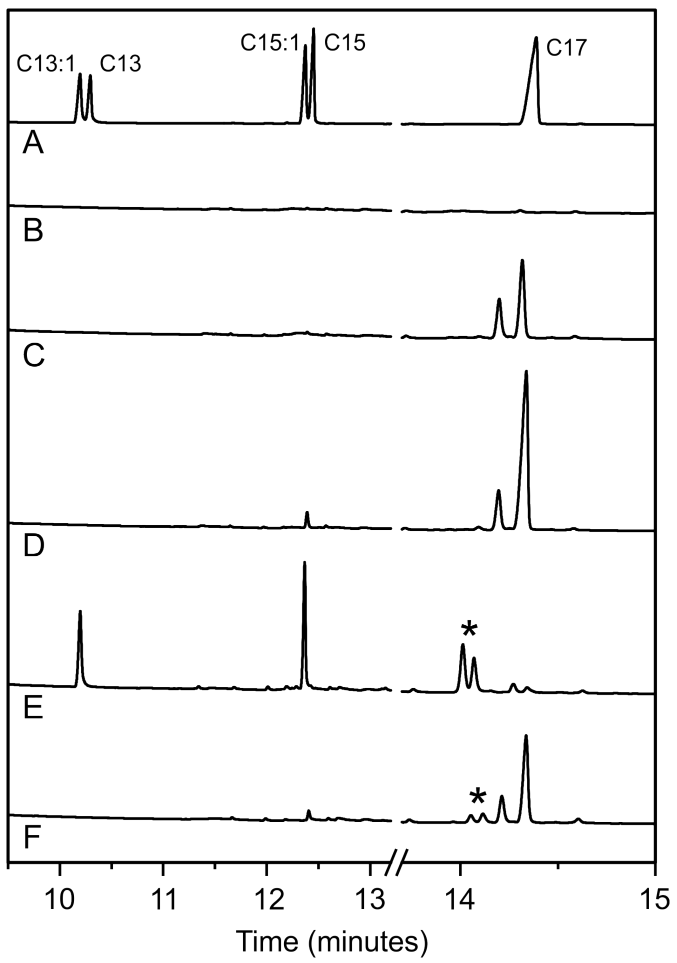

Figure S9. Gas chromatograms of methanolic extracts of S7002ΔoIs substituted with non-native biosynthetic enzymes. All extracts are from strains grown at 38 °C. (A) Authentic alka/ene hydrocarbon standards. (B) 7002ΔOls. (C) ΔOls::6803alk. (D) ΔOls::7942alk. (E) ΔOls::UndA. (F) ΔOls::FAP. The peak for the internal hexadecane standard at 13.4 minutes has been omitted for clarity.

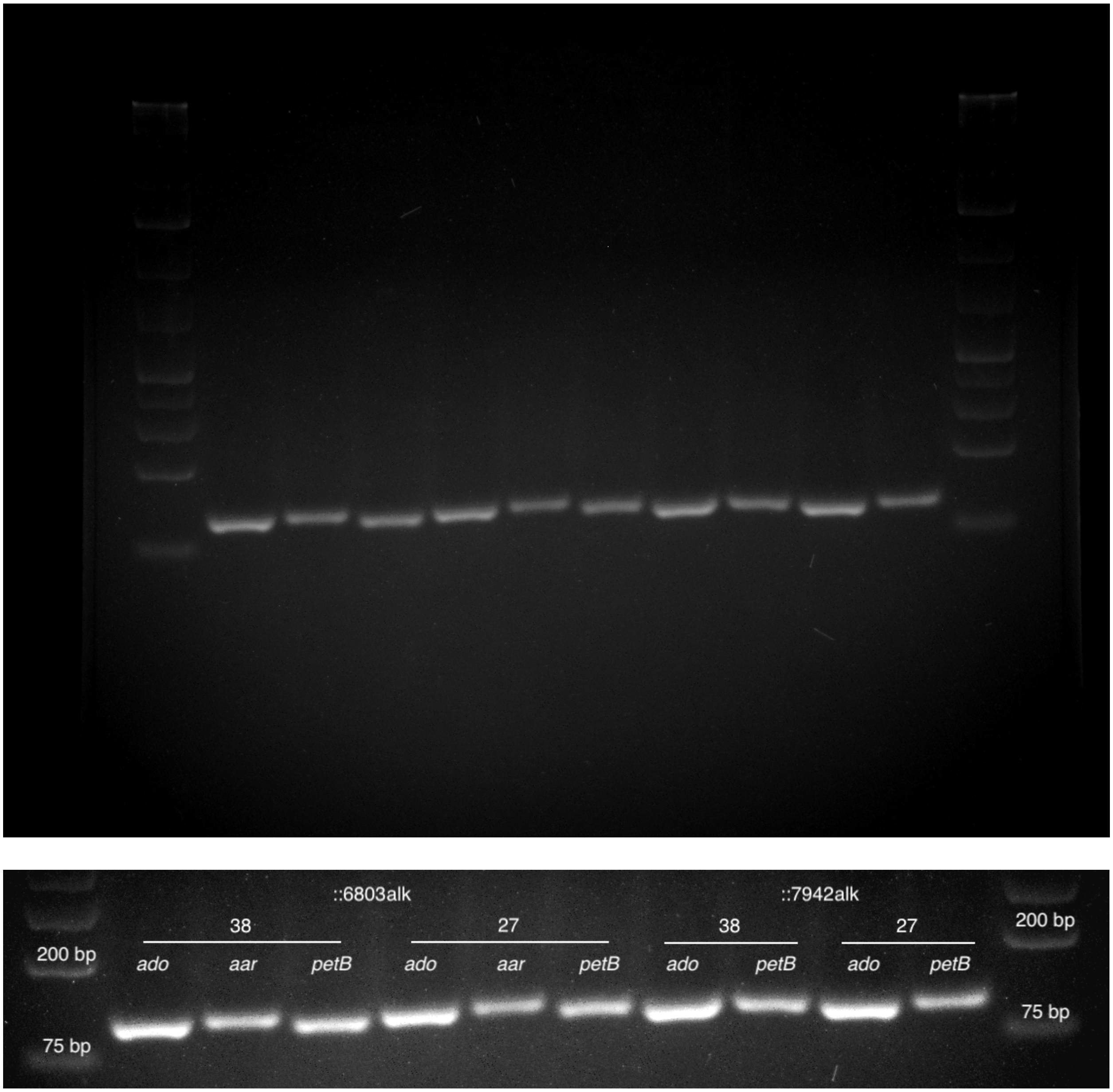

Figure S10. Agarose gel of RT-PCR products from ∆Ols::6803alk and ∆Ols::7942alk strains grown at 38 °C and 27 °C. The top image shows the unmodified gel image. The bottom is a cropped image with the RT-PCR samples labeled. The results show that expression of the S6803 *ado* and *aar* and S7942 *ado* genes is not affected by the temperature change. Expression of *petB* was used an internal RT-PCR control. The outer two lanes contain marker (1 kb Plus DNA Ladder, ThermoFisher).

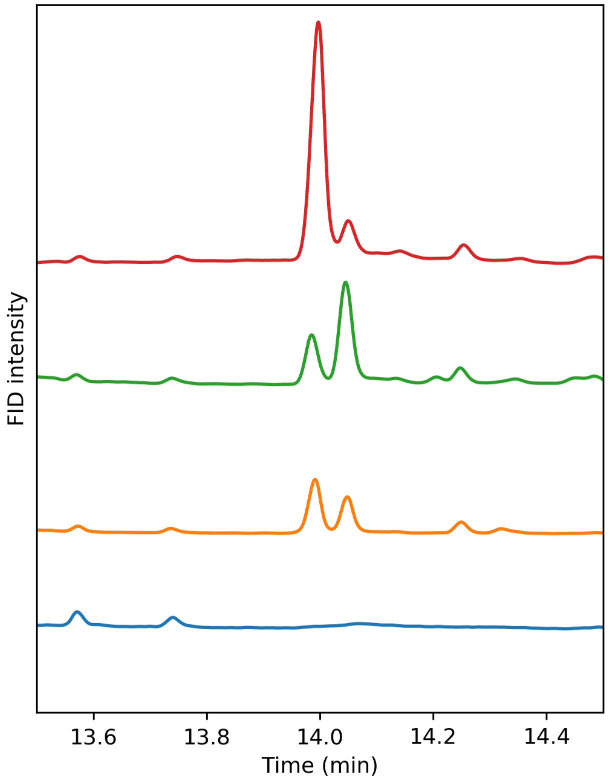

Figure S11. GC traces of organic extracts of ΔOls::UndA cultures grown at 38 °C fed with linoleic acid ($C_{18:2(\Delta 9,12)}$, red trace), α-linolenic acid ($C_{18:3(\Delta 9,12,15)}$, green), and an unfed control (orange). A methanol solvent control plus the two fatty acids is shown in the blue trace. The enhanced production of these two metabolites by ΔOls::UndA in the presence of these fatty acids suggests that these are terminally desaturated heptapolyenes produced by the action of UndA.

Supplementary Tables

Table S1. List of S7002 strain genotypes characterized in this study.

**Strain**	**Strain genotype and gene IDs**
7002::control	pSL3067[Kan^R]
7002::7942alk	pSL3070[*Synpcc7942_1593, Synpcc7942_1594, Synpcc7942_1595*, Kan^R]
7002::6803alk	pSL3071[*sll0208, sll0209*, Kan^R]
7002::trc7942alk	pSL3072[P_{trc1O}-*Synpcc7942_1593, Synpcc7942_1594, Synpcc7942_1595*, Kan^R]
7002ΔOls†	Δ*ols*::Sp^R
ΔOls::control	Δ*ols*::Sp^R, pSL3067[Kan^R]
ΔOls::6803alk	Δ*ols*::Sp^R, pSL3071[*sll0208, sll0209*, Kan^R]
ΔOls::7942alk	Δ*ols*::Sp^R, pSL3258[P_{A2813}- *Synpcc7942_1593, Synpcc7942_1594, Synpcc7942_1595*, Kan^R]
ΔOls::UndA	Δ*ols*::Sp^R, pSL3209[P_{A2813}-*PA14_53120* (F239A), Kan^R]
ΔOls::FAP	Δ*ols*::Sp^R, pSL3244[P_{A2813}-KY511411*, Kan^R]

* Genbank accession number for FAP gene

† The *ols* gene is *Synpcc7002_A1173*

Table S2. List of PCR primers and oligos used in this study.

**Name**	**Sequence 5' – 3'**
Linker1	aggtcgaattcaggtaccat
Linker2	ctagatggtacctgaattcgaccttgca
alk1	ctcctgcaaggtcgaattcaggtaccatgctgacccatgacctcagc
alk2	ccgttgcgctgcccggattacagatcctgccagccatcaggcagtc
alk3	ctcctgcaaggtcgaattcaggtaccatcccagcaacttagactagttttg
alk4	ccgttgcgctgcccggattacagatcctcctaagacagtccctctttgag
alk5	ctcctgcaaggtcgaattcaggtaccatttgacaattaatcatccgg
alk6	gctgcggcatctagtatttctcctctttctc
alk7	agaaagaggagaaatactagatgccgcagcttgaagcc
alk8	tcctgcaaggtcgaattcaggtaccattttagattttctatctagaagaaaaaaat
alk9	gaaaaattcgctcatttgtttttcctcacacattaag
alk10	tgtgaggaaaaacaaatgagcgaatttttcgatc
alk11	ttccgttgcgctgcccggattacagatccttcactccgcgcc
alk12	gcgttctaaagctaaatacatgtagtcgtagctt
alk13	acgactacatgtatttagctttagaacgctgcatgc
alk14	cacgactggtaatactggccatttgtttttcctcacacattaag
alk15	aaaaacaaatggccagtattaccagtcg
alk16	ttccgttgcgctgcccggattacagatcctttaggctgccacggtag
alk17	ctgctcctgcaaggtcgaattcaggtactttagattttctatctagaagaaaaaaa
alk18	gatgagagggagaatgtgggatttggtgtgtatcaatcatattagctcaaaat
eYFP-F	tcctgcaaggtcgaattcaggtaccatcacctgcatcgttacatggtgagcaagggcgag
eYFP-R	ttccgttgcgctgcccggattacagatccttgagttgaaggatcagctcga
Pado-F	tcgaattcaggtaccatcacctgcatcggctgacccatgacctcag
Pado-R	tgaacagctcctcgcccttgctcaccatgagtctgcggtctcctgac
Palk-F	gaattcaggtaccatcacctgcatcgctgttcggacgatggactttc
Palk-R	tgaacagctcctcgcccttgctcaccatgagtctgcggtctcctgac
Ptrc1O-F	ggtcgaattcaggtaccatcacctgcatcgttgacaattaatcatccgg

Ptrc1O-R	ggtgaacagctcctcg
olsUS-F	cgcttgattaccctggac
olsUS-R	gcttaactcaaagtacagagctaaacttcaggcg
spec-F	tgaagtttagctctgtactttgagttaagccgcgc
spec-R	actcaaatcagttgaaacccaagcagcaagcgc
olsDS-F	cttgctgcttgggtttcaactgatttgagtg
olsDS-R	gcgtacccctggtttttc
petB-qPCR1	tcccaaggcaaggagtaacc
petB-qPCR2	ctcaccggtggctttaaacg
ols-qPCR1	cgttgctcccacatagatgg
ols-qPCR2	ttccaggatacagaggcgag
fabH-qPCR1	tggaggtcgccaggataatc
fabH-qPCR2	agtgaactggcggcagag
accB-qPCR1	gagaaacaacgggagtggac
accB-qPCR2	gaactccgtgtgagcaaagg
6803ado-qPCR1	cgcctatagccgcatcaac
6803ado-qPCR2	ccaagcgggtcaactcttc
6803aar-qPCR1	agcggttgctgaagatttagg
6803aar-qPCR2	gccccgtcacacttttcac
7942ado-qPCR1	gcatcaacgcgatcgtgattg
7942ado-qPCR2	ggctagcttgtgaagctcatc

Supplementary Reference

1 Livak, K. J. & Schmittgen, T. D. Analysis of Relative Gene Expression Data Using Real-Time Quantitative PCR and the 2−ΔΔCT Method. *Methods* **25**, 402-408, doi:10.1006/meth.2001.1262 (2001).
